# Disability and all-cause mortality in the older population: evidence from the English Longitudinal Study of Ageing

**DOI:** 10.1007/s10654-016-0160-8

**Published:** 2016-05-13

**Authors:** Benedetta Pongiglione, Bianca L. De Stavola, Hannah Kuper, George B. Ploubidis

**Affiliations:** 1 Department of Medical Statistics, London School of Hygiene and Tropical Medicine, Keppel St, London, WC1E 7HT UK; 2 International Centre for Evidence in Disability, London School of Hygiene and Tropical Medicine, Keppel St, London, WC1E 7HT UK; 3 Centre for Longitudinal Studies, Department of Social Science, UCL Institute of Education, University College London, 55–59 Gordon Square, London, WC1H 0NU UK

**Keywords:** Disability, Mortality, Ageing, Gender

## Abstract

Despite the vast body of literature studying disability and mortality, evidence to support their association is scarce. This work investigates the role of disability in explaining all‐cause mortality among individuals aged 50+ who participated in the English Longitudinal Study of Aging. The aim is to explain the gender paradox in health and mortality by analysing whether the association of disability with mortality differs between women and men. Disability was conceived following the International Classification of Functioning, Disability and Health (ICF), proposed by the WHO, that conceptualizes disability as a combination of three components: impairment, activity limitation and participation restriction. Latent variable models were used to identify domain-specific factors and general disability. The association of the latter with mortality up to 10 years after enrolment was estimated using discrete-time survival analysis. Our work confirms the validity of the ICF framework and finds that disability is strongly associated with mortality, with a time-varying effect among men, and a smaller constant effect for women. Adjusting for demographic, socioeconomic and behavioural factors attenuated the association for both sexes, but overall the effects remained high and significant. These findings confirm the existence of gender paradox by showing that, when affected by disability, women survive longer than men, although if men survive the first years they appear to become more resilient to disability. Sensitivity analyses suggested that the gender paradox cannot be solely explained by gender-specific health conditions: there must be other mechanisms acting within the pathway between disability and mortality that need to be explored.

**Electronic supplementary material:**

The online version of this article (doi:10.1007/s10654-016-0160-8) contains supplementary material, which is available to authorized users.

## Introduction

**Fig. 1:**
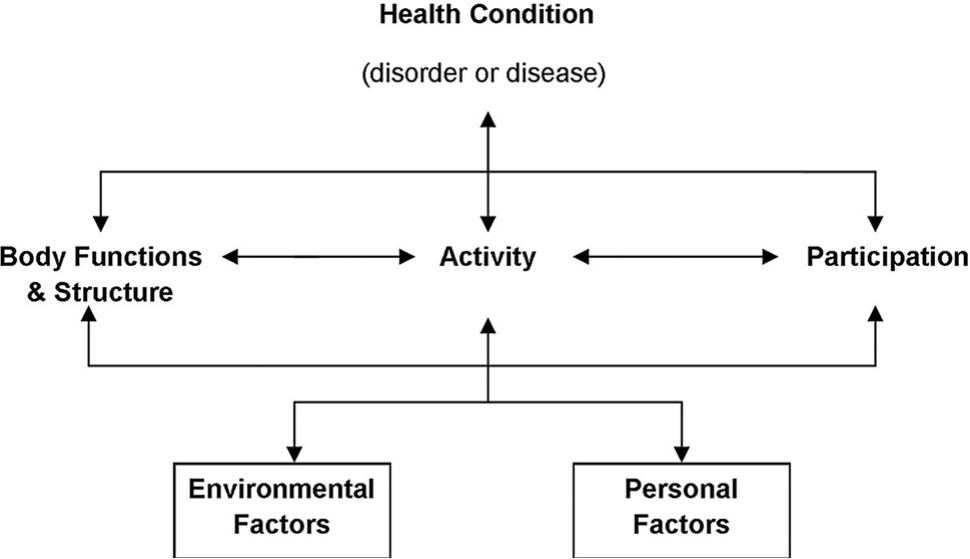
Representation of the International Classification of Functioning, Disability and Health (ICF). *Source*: World Health Organization Geneva 2002, ‘Towards a Common Language for Functioning, Disability and Health: ICF’

In 2001 the World Health Organization (WHO) developed a conceptual framework for describing functioning and disability: the International Classification of Functioning, Disability and Health (ICF). One of the aim of the ICF was to provide a common set of instruments to measure disability to standardize this concept and its use in international studies. The ICF conceives difficulties with human functioning as three interconnected areas (see Fig. 1). This is impairments that are problems in body function or alterations in body structure; activity limitations that are difficulties in executing daily activities such as walking or eating; and participation restrictions that are problems with involvement in any area of life—for example, facing discrimination in employment due to disability [1, p. 5]. Disability refers to difficulties encountered in any or all three areas of functioning.

The ICF is considered the dominant conceptual framework for describing functioning and disability [2]. Nevertheless, it is not yet widely used in research relating or combining disability and mortality. Dale and colleagues [2] examined the relationship between disability and mortality conceiving disability according to the ICF’s framework, focusing on women aged 60–79 years. A key aspect in studying disability and mortality, however, is related to gender differences. The gender paradox in health and mortality is well known in the literature. It was first observed in the mid-1970s [3, 4] and reflects the finding that women live longer than men, but tend to have more disability than males. Many theories have been proposed to explain the ‘gender paradox’ in mortality and disability, among which the most prevalent is that women may have higher prevalence of nonfatal but disabling diseases and men have higher prevalence of fatal and chronic diseases strongly related to mortality. Some researchers [5, 6] hypothesize that higher disability prevalence among women may be a function of longer survival in disability rather than higher incidence of disability.

With our work we seek to contribute to the debate of the gender paradox in health and mortality by (1) showing whether the association between disability and mortality differs between men and women (2) proposing possible explanations of why it may occur. More specifically, we measure disability among the older population using data from the English Longitudinal Study of Ageing (ELSA), and empirically test with a measurement model the construct validity of the WHO’s ICF. Based on this comprehensive interpretation of disability, we then apply discrete-time survival analysis (DTSA) to study the impact of disability measured at baseline on mortality observed over the course of a decade, and assess whether and how this association changes over time, stratifying the analysis by gender.

## Materials and methods

### Data source and sample

This study used data drawn from the first wave of the English Longitudinal Study of Ageing (ELSA), which took place in 2002/2003. Briefly, ELSA core members are a representative sample of the noninstitutionalized population, living in England, who were aged 50 years or older at the time of interview. 11,391 core-member respondents were recruited at wave 1. For our analysis, we included all participants who had complete records on all disability items, leaving us with a sample of 9715. At the time of interview, respondents were asked to give their permission to link their data to the National Health Service Central Register (NHSCR) mortality records. For those who gave their consent, information on mortality was available by year from 2002 to 2011. Interviews were done using computer-assisted interviewing and self-completion questionnaires.

### Measures

#### Death

The primarily outcome of this analysis was deaths occurred from 2002 to 2011. As time of death was available only by year, binary time-specific event indicators were created for each period of observation (ten intervals). For some respondents (n = 358) status of death was available but time of death was unknown; in this case information were partially retrieved looking whether respondents took part in the following surveys; if they were interviewed in later waves, they were assumed to be alive at least until the year of the last survey they responded; otherwise they were considered lost to follow-up and their event indicators treated as missing. This way three patterns of observations were possible: (1) survivors or censored: individuals who did not experience the event and were followed-up for all time-periods of observation; (2) dead: individuals who experienced the event at some point during the period of observation; (3) lost to follow-up: individuals who dropped out the study before it ended.

#### Disability

Variables describing disability were selected according to the WHO’s ICF framework, in order to construct the impairment, activity limitation and participation restriction components. Consulting the WHO’s ICF browser, one author selected all possible disability items from the questionnaire to be included in the measurement model; the list was screened in agreement with another author and selected items were classified in a double-blind fashion in one of the three components; in case of disagreement a third opinion was sought for the final classification. Inter-rater agreement for classification of selected items was measured using the kappa statistic [7]. A total of fifty items were selected from the questionnaire to construct the ICF model: 19 for impairment, 20 for activity limitation and 11 for participation restriction (Supplementary Table 1). Impairment was described by variables such as self-rated eyesight and hearing, chronic conditions such as high blood pressure and arthritis, and questions about pain. Activity limitation was assessed by questions on ADLs and mobility functions, for example climbing flights of stairs or walking 100 yards. Finally, participation included questions on instrumental activities of daily living (IADLs), and various limitations due to health problems, such as using public transports or working. Variables were all either dichotomous (i.e. yes/no answer) or ordered categorical, for example ranging from ‘excellent’ to ‘poor’, from ‘never’ to ‘always’ and from ‘no difficulty’ to ‘unable’. A list of the questions asked for each item and possible answers is available in the appendix (Supplementary Table 1).

#### Confounders

A number of potential confounders known to be related to disability and mortality from the literature (see for example [8–13]) were accounted for in the survival models. These included basic demographic characteristics, such as age at wave 1, marital status and household size; socioeconomic position (SEP) measured through education, income, wealth and occupation; socioeconomic background represented by father’s occupation when respondent was 14; health-related behaviours including smoking, drinking and physical activity; and presence of limiting long-lasting illness. In sensitivity analyses, objective measures of health were also introduced as additional confounders in the analyses that used the information collected at wave 2 (2004/2005) where health measures were assessed during the nurse visit with survivors up to that wave included in the analysis. Four observer-measured indicators were selected. These were blood assays for inflammation, blood clotting and cholesterol—all known to be associated with risk of heart disease- and a measure of respiratory functioning. The inflammatory activity in the body was measured by the level of C-reactive protein (CRP); blood clotting by a protein called fibrinogen; cholesterol is a type of fat present in the blood and was assessed as total cholesterol. Respiratory functioning was measured by Forced Vital Capacity (FVC), which is the volume of air that can forcibly be blown out after full inspiration; three measurements were taken of FVC, and we used the highest technically satisfactory reading.

### Analysis

The analysis was carried out in two steps. First we estimated factor scores for disability using a latent variable model, then we used the stored factor scores in survival analysis.^1^

#### Measurement model

For the first step, a three factor first-order model was first fit to assess the ICF structure using the items selected for each ICF component, i.e. impairment, activity limitation and participation restriction.

Since all observed items were either categorical or binary, the fitted model can be formulated as follows. Categorical/binary observed indicators (y_ij_) are related to continuous latent variable (η_j_) via a normal ogive response model, such that:

1 $$y_{ij} = \left\{ {\begin{array}{*{20}l} 1 \hfill & {{\text{if}}\;y_{ij}^{*} > \tau_{i} } \hfill \\ 0 \hfill & {\text{otherwise}} \hfill \\ \end{array} } \right.$$

where $y_{ij}^{*} = \beta_{i} +\uplambda_{i}\upeta_{j} + \varepsilon_{ij}$ for i = 1, …, I_j_ (I_j_ being the number of observed indicators for latent variable j) and j = 1, …, J (J being the number of individuals). We also assume that

$$\upeta_{\text{j}} \sim\,N(0,\sigma^{2} ),\;\upvarepsilon_{\text{ij}} \sim\,N\left( {0,1} \right),\;{\text{covariance}}\;\left( {\upeta_{\text{j}} ,\upvarepsilon_{\text{ij}} } \right) = 0$$

where $\sigma^{2}$ is the variance of the latent measure. For simplicity, here we refer to unidimensional model; for more general notation see Rabe-Hesketh and Shrondal [14].

Model (1) can be equivalently expressed as:

$$\Pr \left( {y_{ij} = 1|\eta_{j} } \right) = \Pr \left( {y_{ij}^{*} > \tau_{i} |\eta_{j} } \right) =\Phi \left( {\beta_{i} + \lambda_{i} \eta_{j} } \right)$$

$$\Phi ^{ - 1} \Pr \left( {y_{ij} = 1|\eta_{j} } \right) = \beta_{i} + \lambda_{i} \eta_{j}$$

where Φ(·) is the cumulative standard normal distribution and Φ^−1^ is the probit link.

Modification indices (MIs) were examined to improve model fit. MIs quantify the decrease of the χ^2^ goodness of fit measure when the corresponding parameter is freed; they indicate whether any of the observed items should be correlated above and beyond their assumed relationships with latent factors. As this test’s recommendations are directly motivated by the data and not by theoretical considerations [15, p. 491], we used them to suggest improvements but did not tie model specification on their values.

**Fig. 2:**
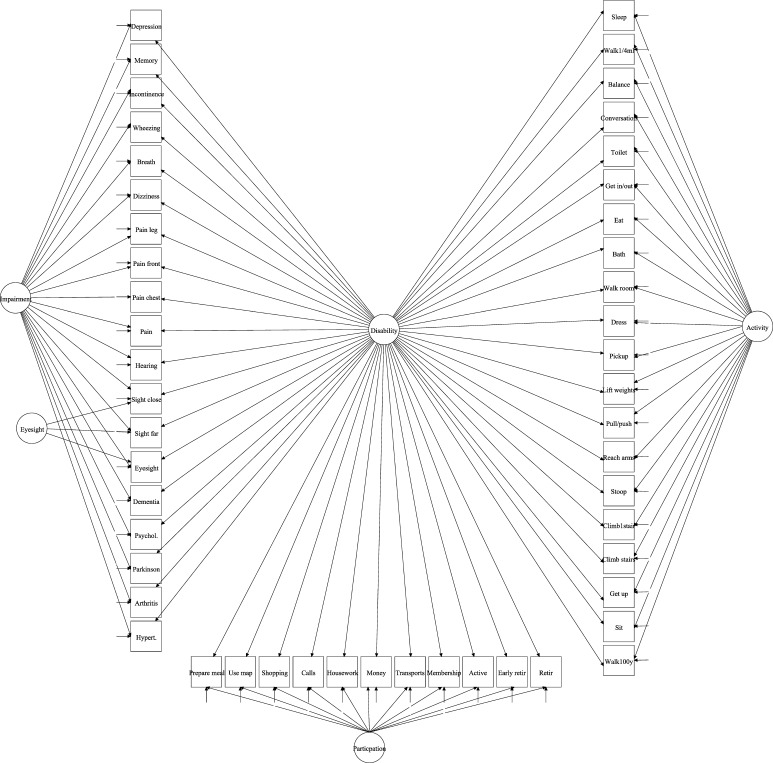
General-specific measurement model. Names of observed variables (*rectangles*) are those listed in Table 1

The best fitting first order model that reflects the ICF structure described impairment, activity limitation and participation restriction and was improved by adding an extra factor for eyesight within the impairment component. Based on this construct and reflecting the WHO conceptualization, we fitted a second order model, where disability was the second order factor and impairment, eyesight, activity limitation and participation restriction were the first order factors. However the model presented some inconsistencies.^2^ To deal with that, we decided to conceptualize disability in a general-specific model where the observed items are explained by one general factor disability- and domain-specific factors (see Fig. 2). Both the general and the specific factors were linked to the observed items as described above, and all factors were assumed to be uncorrelated with each other.

For identification purposes, both models (first order and general-specific) were defined constraining all factor variances to be equal to one, and allowing the error terms of the manifest items ‘pain in chest’ and ‘pain’ to correlate. Model estimation was performed using only complete records via weighted least squares means and variance adjusted (WLSMV) [16].^3^

Model fit was assessed using the Root Mean Square Error of Approximation (RMSEA) which assesses absolute fit, and two comparative indices, Comparative Fit Index (CFI) and Tucker–Lewis Index (TLI), which compare the model with the unrealistic null model of uncorrelated items. Fit is typically considered ‘good’ if the RMSEA is below 0.05 and the CFI and TLI are above 0.90 [14, p. 86].

#### Discrete-time survival analysis (DTSA)

Data were set in a way to carry out DTSA in a general latent variable framework [17]. A binary time-specific event indicator was created for each of the ten time periods, with the probability of an event occurring during an interval denoted by h(j), j = 1, …, 10, and referred to as the hazard probability for that time period [17].^4^ The first step was to fit a crude mortality risk model that included the 10 binary time-specific event indicators of death,^5^ with no predictors (including no intercept) or in other words to estimate the interval-specific risks (i.e. the probabilities for each time interval, analogous to separate intercepts in a regular regression model).

These probabilities were then related to covariates through a logit link function—that is, logistic regression—so that the effect of a covariate on the timing of death is parameterized by its effect on the log odds of an event during a given time interval [18]. For a single covariate $x$, its effect on the probability of event occurrence in period j is expressed in terms of the log odds ratio (log OR) β_j_^6^_:_

$${\text{logit}}\;h\left( j \right) = \log \left( {\frac{h\left( j \right)}{1 - h\left( j \right)}} \right) = - \tau_{j} + \beta_{j} x$$

$$h\left( j \right) = \frac{1}{{1 + { \exp }\left( {\tau_{j} - \beta_{j} x} \right)}}$$

Then, we evaluated whether the corresponding logORs were constant over the 10 intervals (i.e. β_j=_ β for all j, equivalent to the proportionality assumption), separately for each of the covariates, by introducing each covariate in the model (i.e. assuming a time invariant effect) and then including an interaction between the covariate and time (i.e. allowing for time varying effects) tested, using the log-likelihood ratio test (LRT). For disability, we double-checked whether its effect was time-varying controlling first only for age and then for the complete set of selected confounders.

Finally, we fitted models that includes the confounders sequentially, by group. In the baseline model we considered the effect of disability on mortality without controlling for any confounders but age; and in the full model all potential confounders were added, including long-lasting illness and health-related behaviours (all measured at wave 1).

Events indicators were treated as missing in correspondence of time intervals that followed the time when the event occurred or when the individual was lost to follow-up. Missingness was assumed to be at random (MAR) which for this model corresponds to uninformative loss to follow-up; FIML estimation with robust standard errors (MLR) was used [17]. When we added confounders, we incurred in missing values for these $x$ variables; however only 4 % of data were missing, corresponding to three main missing patterns. When confounders were added into the model, complete case analysis (CCA) was carried out. However, this way adjusted for age analyses and adjusted for all confounders analyses were based on different numbers of observations; to deal with this problem, we first repeated the age adjusted models on the same numbers as those for the fully adjusted analyses, and secondly we the fully adjusted model was re-run using FIML in order to have the same sample size as in age adjusted models. Details on missing data patterns and results for CCA and for regressions using FIML are provided in the appendix (Supplementary Tables 2 and 3).

#### Sensitivity analysis

A number of robustness checks were implemented in order to assess whether gender differences in the association between disability and mortality were driven by gender differences in prevalence of specific disabling diseases. In the first instance, we accounted for the fact that self-reported measures of health may not capture specific diseases and there may be a gender effect in the probability of reporting health limitations. To account for this potential bias, observer-measured health indicators were additionally considered as potential confounders. To this aim, we replicated the analysis including only respondents interviewed at wave 1 who took part in the following survey and using information on physical conditions measured during the nurse visit at wave 2. Four observer-measured indicators were selected and added as confounders in DTSA based on data from wave 2.

With the same rationale, but using a different approach, we also re-estimated the measurement model for disability dropping the items describing health/body functions (i.e. hypertension, arthritis, dementia, Parkinson, psychological problems and depression) originally included within the impairment component, to make sure that differences in mortality were not led by body functions and structures whose prevalence is more likely to differ between men and women.

To test whether the measurement model differed for males and females, we also re-estimated the factor scores for disability running separate analyses for men and women, and then testing whether there was heterogeneity by sex (we used a multiple group analysis for the total sample assuming strong invariance). The survival analysis model was also refitted using these new disability scores.

Finally, to account for possible differences across age groups, the original measurement model—as described in the previous paragraph—was re-estimated via multiple group analysis, without stratifying by gender. Then, we run DTSA using the resulting disability factor score and stratifying the sample by age group (i.e. 50–64, 65–74, 75+). Additionally and separately, we also re-run DTSA including an interaction term for age and disability (as measured in the baseline model).

## Results

### Sample

Of the 9715 respondents 46 % were men (4455) and 54 % women (5260). Over the course of the study, 21 % of male and 16 % of female respondents died (Supplementary Table 4). Demographic and socioeconomic characteristics are shown in the appendix (Supplementary Table 4). In general, demographic characteristics were quite similar between females and males; the average age of men and women was 64.4 and 64.8 years respectively with more women than men being aged 75+ (19.5 % of females compared to 17.4 % of males); higher proportions of women were widowed as expected due to their longer life expectancy. Men reported higher SEP in all indicators, e.g. higher education, income, occupational class. On the other hand, women had healthier behaviours, reporting higher proportions in those that never smoked as well as lower percentage of heavy drinkers. Finally, among respondents survived at wave 2, men had a more healthy profile than women with regards to all biomarkers and almost same level of inflammation.

### Measurement model

The final agreed list of disability variables (kappa statistic for inter-rater agreement equal to 0.85) consisted of 50 items (19 impairments, 20 activities and 11 participations—Supplementary Table 1). The prevalence of these variables was higher for women than men (Table 1), with the exception of difficulty in communicating (conversation) and being engaged in social activity (active), and to a lesser extent in visual functioning. Descriptive statistics show that more men than women died, but women overall had more disability problems than men at baseline.

**Table 1:** Prevalence of health indicator by gender

Disability items	Men %	Women %
Hypertension	36.6	38.6
Arthritis	25.5	38.1
Parkinson	0.4	0.4
Psycho problems	5.8	8.8
Dementia	0.4	0.2
Self-rated eyesight (less than good)	12.8	15.5
Eyesight at distance (less than good)	6.4	9.8
Eyesight close (less than good)	9.9	10.8
Hearing	23.8	28.4
Troubled with pain	34.1	40.5
Pain in chest	33.3	27.4
Pain across the front of chest	11.5	7.5
Pain in leg	28.3	30.6
Dizziness	11.8	16.5
Shortness of breath	32.1	42.5
Shortness of breath with wheezing	14.5	14.8
Incontinence	8.3	20.8
Self-rated memory (less than good)	32.3	30
Depression	14.1	18.3
Walking 100 yards	11.4	11.1
Sitting for 2 h	13	14.8
Getting up	22.2	28.1
Climbing stairs	28.8	41.2
Climbing 1 flights of stairs	11.6	15.3
Stooping	31.1	38.4
Reaching arms	9.2	12
Pulling/pushing	12.3	20.8
Lifting weights over 10 lb	15.9	31.9
Picking up 5p coin	4.5	5.2
Dressing	14.1	11.4
Walking across room	2.6	2.8
Bathing	9.9	12.5
Eating	1.3	1.8
Getting in/out bed	6	6.3
Toileting	3	3.1
Following conversation	40.8	28.1
Keeping balance	18.5	24.9
Walking quarter mile	25.1	28.9
Restless sleep	34.7	45.3
Preparing hot meal	3.4	3.7
Using map	2.2	6.2
Grocery shopping	6.1	9.9
Making calls	1.8	0.9
Housework	13.2	16.4
Managing money	1.9	1.6
Using transports	5.4	7.9
Being member of any org.	28.8	32.1
Doing activity	34.5	22.9
Early retirement (due to health)	7	3.7
Retirement (due to health)	3.1	4.8

Following this classification, a latent variable model appropriate for the nature of the indicators was implemented. A first-order multidimensional model was first estimated, and its fit was rather poor (see Table 2). Some items presented high modification indices both for factor loadings and covariances among measurement errors. In particular, eyesight items (which are self-rated eyesight, being able to seeing at distance and close) presented high modification indices for both factor and covariances among their measurement errors. Rather than allowing the errors of the eyesight items to correlate, we introduced within the impairment factor an extra eye-specific latent factor to explain eye-items variability, producing a sort of general-specific model within the multidimensional first order model. The resulting model fit was highly satisfactory (Table 2). Standardized factor loadings λ_ij_, which express the strength of the association between the indicators and latent variables, by rule of thumb are considered satisfactory when |λ_ij_| > 0.4 [19]. Standardized factor loadings obtained from the first-order model showed that 13 out of 19 indicators of impairment were strongly associated with this factor; 19 out of 20 indicators of activity were strongly associated with this factor and 8 out of 10 indicators with participation factor. Particularly high were the factor loadings for activity, in most cases larger than 0.75 (Supplementary Table 5).

**Table 2:** Goodness of fit

Model	CFI^a^	TLI^b^	RMSEA^c^
(1) First order model (3 factors)	0.873	0.867	0.067
(2) Fist order model (3 factors + eyesight component)	0.945	0.942	0.042
(3) General-specific model	0.956	0.952	0.039

^a^Comparative Fit Index

^b^Tucker–Lewis Index

^c^Root mean square error of approximation

**Fig. 3:**
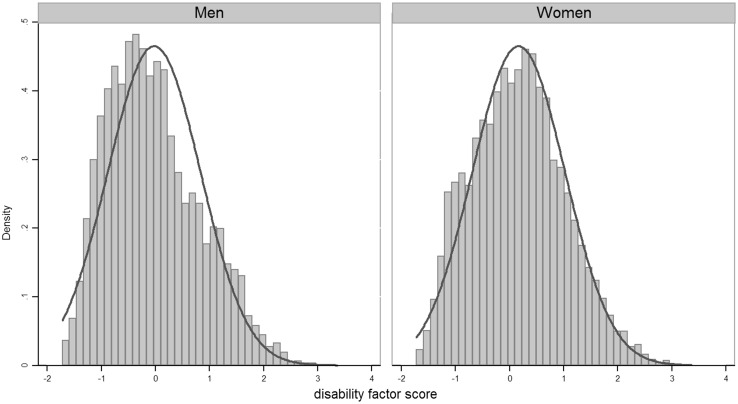
Disability factor score by gender

Based on the first-order measurement model described above, a general-specific model was fitted to identify the latent disability structure (Fig. 2). Goodness of fit (GoF) indicators are presented in Table 2. The distribution of the disability factor score, derived from the general-specific model, is shown in Fig. 3, by gender. The distributions are approximately Gaussian (Fig. 3); with that for males slightly more right-skewed than that for females, meaning that, compared to women, fewer men had high disability score. The average score of disability was higher for women; on a range going from −1.72 to 3.36, the female average score was 0.165, whilst on a range from −1.72 to 2.88 the male average score was equal to −0.025, i.e. 0.19 units lower (*p* value <0.001). When controlling for various chronic conditions not included in disability measure and for self-reported long-lasting illness, the mean difference in disability between women and men remained the same (0.19, *p* value < 0.001).

### Discrete-time survival analysis

**Fig. 4:**
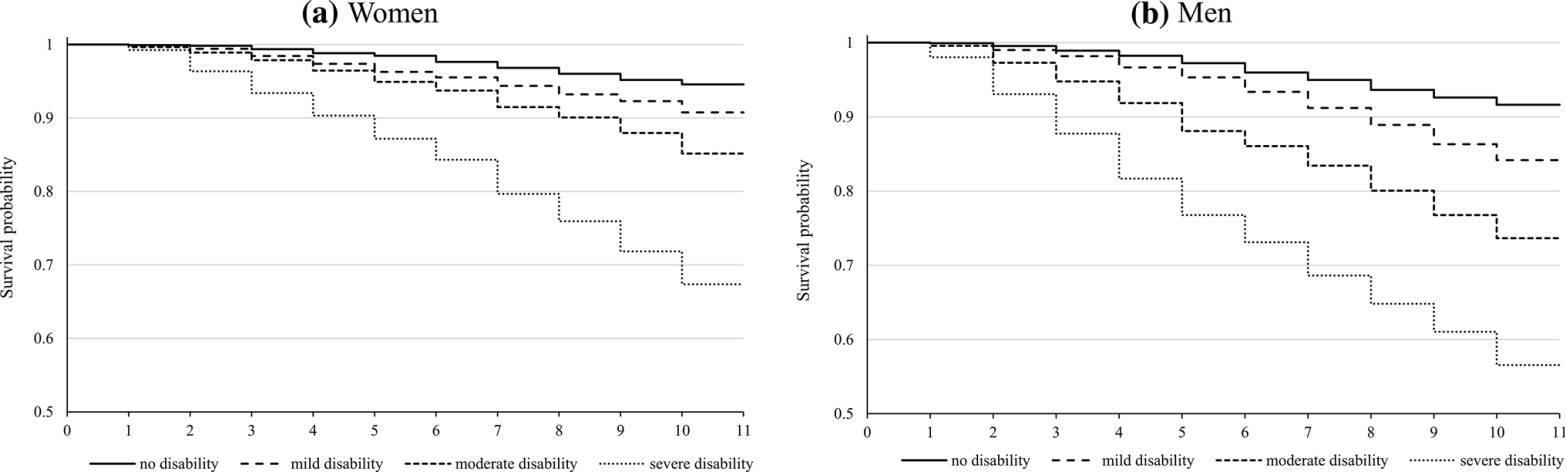
Kaplan–Meier survival estimate, by disability component and gender. Results are presented by gender but disability factor score is estimated for the pooled sample

1775 respondents died over the course of the observation period, 53 % were men and 47 % women. Overall, mortality rate was 0.56 % for men and 0.34 % for women in the first interval (first year of follow up since 2002) and almost 3 % in the last interval (3.1 and 2.9 % for men and women respectively), with a relatively steadily increasing trend during the observation period. The Kaplan–Meier survival curve in Fig. 4 illustrates the survival curves by quartile of disability, separately by gender. The estimated survival curves are lower as the severity of disability increases, both for women and men. Male disadvantage in mortality is observed across each disability quartile and widens over time; the gap in mortality between men and women is more pronounced for the two most disabled groups. In particular, 56.5 % of men having the highest disability level survive to the end of the 10-year period, while the equivalent survivors percentage for women is 67.4 %.

To evaluate whether the effect of the pre-defined confounders on mortality were time-varying we introduced in the model each variable separately with/without its interaction with time, while controlling for age. The constant proportional hazard assumption (i.e. time-invariant effect) was rejected for age and physical activity, but the latter only for men (Supplementary Table 6 for LRT test results). To assess the proportionality assumption for the predicted disability score we performed separate LRTs for its interaction with time, first controlling only for age, and then adjusting for all confounders. In both cases, disability was found to have time-varying effects for men and a time-invariant effect for women.

The parameter estimates for disability (expressed on the odds ratio scale) are shown in Table 3. For men, the time-specific disability odds ratios estimated controlling only for age (Model 1) were all significantly greater than 1, albeit decreasing over time. Although we did not observe a continuously declining trend, the test for trend showed evidence of a linear trend ($X_{8}^{2}$ = 17.54, *p* value = 0.025). The estimated disability OR corresponding to the first time period (2002) was 3.4 (95 % CI 2.12, 5.38), which means that for one-unit (1 SD in the latent score) increase in disability score the expected increase in the odds of mortality was by a factor of 3.4. Over subsequent time intervals the estimated ORs declined, but remained significantly higher than 1. Interestingly the estimated ORs dropped substantially immediately after the first period, from 3.4 to 2 in the following period; then the decline became more gradual. With regards to women, as we did not reject the proportionality assumption, the disability effect on mortality was estimated assuming a time-invariant effect, leading to a single estimated OR of 1.65 (95 % CI 1.51, 1.81; Model 1).

**Table 3:** Disability odds ratios for mortality

	Time interval since disability measurement (years)	Model 1^a^		Model 2^b^	
		OR^c^	95 % CI^d^	OR^e^	95 % CI^d^
Males	1	3.381***	(2.12; 5.38)	2.237***	(1.27; 3.95)
	2	2.038***	(1.55; 2.69)	1.789***	(1.27; 2.52)
	3	2.157***	(1.68; 2.76)	1.875***	(1.39; 2.54)
	4	2.114***	(1.69; 2.65)	1.424***	(1.09; 1.86)
	5	1.826***	(1.45; 2.3)	1.445***	(1.09; 1.91)
	6	1.296*	(1; 1.69)	1.04	(0.77; 1.4)
	7	1.557***	(1.22; 1.98)	1.304*	(0.98; 1.74)
	8	1.499***	(1.18; 1.9)	1.305*	(0.99; 1.72)
	9	1.571***	(1.24; 1.99)	1.375**	(1.04; 1.82)
	10	2.083***	(1.62; 2.67)	1.955***	(1.46; 2.62)
Females	Time-invariant effect	1.654***	(1.51; 1.81)	1.365***	(1.21; 1.54)

^*^ *p* < 0.1; ^**^ *p * < 0.05; ^***^ *p * < 0.01

^a^Model 1: model adjusted for age only

^b^Model 2: fully adjusted model: adjusted for age, demographic and socioeconomic confounders, father’s occupation and long-lasting illness

^c^Test for linear trend χ^2^(8) = 17.54, *p* value = 0.025

^d^SE estimated from pooled logistic regression

^e^Test for linear trend χ^2^(8) = 15.96, *p* value = 0.043

Table 3 also reports the estimated disability odds ratios by gender, obtained from fitting the model fully adjusted for demographic, socioeconomic and behavioural factors, father’s occupation and limiting long-lasting illness. For men, the estimated disability OR for time interval 1 decreased from 3.4 in the age-adjusted model to 2.2 in the fully adjusted model. The effect of confounders seemed particularly strong in this first interval, and although the estimated ORs in the following intervals were all smaller compared to those of model 1, they were all significant (at 5 % significance) with the exception of those for interval 6, 7 and 8. Among women, the estimated time-invariant effect of disability on mortality moderately declined after controlling for confounders, dropping from 1.65 to 1.36 (95 % CI 1.21–1.54). As a sensitivity analysis we also checked for a moderating effect of age and found a significant interaction of age and disability for men, such that the impact of disability measured at baseline becomes smaller as men age, while for women the interaction was not significant. When stratifying the analysis by age group, after age 75 the results for men disappear and disability OR decreases across age groups for women only (Supplementary Table 7).

When observer-measured health indicators were considered as potential confounders, DTSA was performed using the respondents interviewed at wave 1 who took part in the following survey, which was nurse-led and included collection of biomarkers. The results are shown in Table 4. The fully adjusted model was replicated first (columns 1 and 2), and then inflammation, blood clotting, cholesterol and respiratory functioning were added among the confounding variables (column 3 and 4). Among women the time-invariant effect of disability on mortality slightly decreased when controlling for observer-measured health indicators, whilst for men the estimated time-varying effect of disability was no longer significant both when adjusting or not adjusting for the biomarkers. (The results of other sensitivity analyses are not presented here, but available in the appendix and commented in the discussion section).

**Table 4:** Disability odds ratios for mortality, wave 2

	Time since disability measurement (years)	Model 1^a^		Model 2^b^	
		OR	95 % CI^c^	OR	95 % CI^c^
Males	1	2.403*	(0.97; 5.94)	2.316*	(0.93; 5.77)
	2	1.649*	(0.95; 2.87)	1.598	(0.91; 2.8)
	3	0.985	(0.58; 1.66)	0.952	(0.56; 1.61)
	4	1.559*	(0.92; 2.64)	1.519	(0.89; 2.58)
	5	1.343	(0.8; 2.25)	1.297	(0.77; 2.18)
	6	1.151	(0.69; 1.92)	1.114	(0.66; 1.87)
	7	0.821	(0.52; 1.29)	0.796	(0.51; 1.25)
	8	1.48	(0.8; 2.74)	1.468	(0.79; 2.72)
Females	Time-invariant effect	1.435**	(1.12; 1.83)	1.331**	(1.04; 1.71)

^*^ *p* < 0.1; ^**^ *p* < 0.05;^ ***^ *p* < 0.01

Sample size males = 1897; females = 2162

^a^Fully adjusted model

^b^Fully adjusted model + observer-measured indicators

^c^SE estimated from pooled logistic regression

## Discussion

Our study provides evidence on the association between mortality and disability in the older population and how this differs between men and women. Consistent with previous research, survival was found to be higher for women than men, whereas women had higher prevalence of disability. When looking at the relationship of disability at baseline with mortality observed over a decade later, the present study revealed: (1) increasing odds of dying as the baseline disability score increased, both for women and men with the association being stronger among the latter; and (2) decreasing association over time for men, as the impact of baseline disability on their mortality decreased with longer survival; (3) no variation over time for women, as the effect of disability remained constant over the 10-year period of observation.

With regard to men, the most striking result was the dramatic drop in the effect of disability on mortality from baseline period to the following year (2.2–1.8 per 1 standard unit change in disability score): disability in men, compared to women, seemed to have a stronger association with mortality in the very short rather than in the long term, when their estimated ORs converged to those in women. This could mean that men become more resilient to disability the longer they survive, and therefore that the effect of disability on their mortality in the long-run becomes less pronounced. Alternatively it could mean that disability is measured differently in men and women. However, as discussed in the next paragraphs, when we investigated this by extending the disability measurement model we found no evidence to support this explanation. For women, the impact of disability was found to be constant over time and overall the effect was smaller than that experienced by men. This is in accordance with the gender paradox in morbidity and mortality, and shows that in fact women spend a higher proportion of their life in disability because they survive longer with disability, suggesting that higher disability prevalence among women may be a function of longer survivorship with disability rather than higher incidence of disability.

Along with evidence confirming the existence of the gender paradox among the English population aged 50+, we sought possible explanations of why it may occur. To address this question, we adopted three different strategies, whose results are discussed below. (1) In this study, we interpreted disability as a general phenomenon that may affect men and women to a different extent, rather than intend gender differences in disability depending on the definition of disability itself. Accordingly, disability was measured on the pooled sample. To investigate whether gender may instead affect the measurement itself of disability, we replicated the latent variable measurement model considering men and women separately and also running a multiple group analysis in the pooled sample (results are presented in Supplementary Table 8). The resulting latent measure of disability was in both cases substantially similar to the results obtained from the pooled sample and results of DTSA were the same as those obtained in the original model. This suggests that the different impact of disability on mortality for men and women does not depend on gender-specific features of disability. (2) Additionally, since men are known to suffer more than women from fatal conditions, such as heart disease, and these conditions may not be captured by self-reported indicators, we also considered the confounding effect of observer-measured health indicators (measured at wave 2). We expected that after controlling for these indicators the effect of disability on mortality would decrease and the drop to be larger for males than females. Among women disability continued to exert a similar effect, while for men we found no evidence of an association between disability and mortality at wave 2. This discrepancy of results between sexes might be explained by the fact that the sub-sample of survivors to wave 2 was likely to be different for men and women, with the male sub-sample consisting of a more highly selected—less disabled—group than the equivalent females. Differences in terms of survival between men and women were not unexpected. What is surprising is that the consequences of male disadvantage in mortality and advantage in disability were visible already after 2 years from the beginning of the observation. (3) Finally, we also re-estimated the general-specific model for disability dropping some impairment items that described health functions, to make sure gender differences in mortality were not led by body functions and structures that may affect men and women differently. Again, the latent measure of disability obtained dropping these variables was very similar to the one obtained in the original measurement model, and the results of DTSA (Supplementary Table 9) essentially depicted the same patterns found using the original measure of disability. All the sensitivity analyses suggest that the observed differences in the association between disability and mortality in men and women are not driven only by gender-specific health conditions and body structures.

A complementary objective of the study was to provide a comprehensive definition of disability in order to test empirically the construct validity of the WHO’s ICF framework when applied to the older population. After explorative investigations, disability was conceived as a general independent factor, and impairment, activity and participation as separate specific factors. The results of our study suggest that the three ICF components can be detected using the questions asked in ELSA, and indeed the first order factor model had a good fit. When it came to relate these parts with the concept of disability, disability was conceived as a single construct common to all individual indicators, explaining some proportion of their covariation; while the specific domains, i.e. impairment, eyesight, activity limitation and participation restriction, explain additional covariation among observable indicators. Detailed explanation of why we chose a general-specific model, may be found in the appendix (Supplementary Material B).

Finally, we highlight the strengths and weaknesses of this work. Strengths of the study include the availability of representative of the older population of England longitudinal dataset and the availability of various disability indicators that allowed us to reliably capture the ICF conceptualisation of disability. On the other hand some potential limitations should be considered while interpreting our results. There were no questions on the onset of disability, therefore it was not possible to estimate how long respondents survived from the actual disability onset. However, adjusting for pre-existing long-lasting limiting illness accounted, at least in part, for pre-existing disability; and this enabled us to consider the effect of disability at baseline (wave 1) on mortality as independent from any pre-existing disability/illness. A key point of this study, which represents both a strength and limitation, was that disability (and all confounders) was only measured at the study onset. This way, we did not know how disability had already impacted on health and mortality nor how it evolved over the observation period. This limited our understanding of its relationship with mortality. Nevertheless, the baseline effect can still be interpreted net of any effect that disability change over time on mortality might have had. Moreover, one of the advantages of measuring disability and all confounders at baseline is that, while keeping the model simple, we do not incur reverse-causality problems. Another limitation—as in most observational studies—is bias due to unmeasured confounders and/or residual confounding that might still bias the association under study. We acknowledge this as a potential source of bias, although we believe the most relevant confounders were taken into account.

## Conclusion

The present work contributes to the debate on the gender paradox in health and mortality by showing that women spend a larger proportion of their life in disability because they survive longer with disability. We also enrich the discussion on possible explanations of why this occurs and show that gender differences in the association between disability and mortality are not driven only by gender-specific health conditions and body structures. There must be some other mechanisms acting within the pathway between disability and mortality that make women survive with disability better than men. Future studies should focus on exploring these mechanisms to fully understand the gender paradox in health and mortality.

## Electronic supplementary material

Below is the link to the electronic supplementary material.

Supplementary material 1 (DOCX 39 kb)

## Abbreviations

ADLs: Activities of daily living
DTSA: Discrete-time survival analysis
IADLs: Instrumental activities of daily living
ICF: International Classification of Functioning, Disability and Health
LRT: Likelihood ratio test
SEP: Socioeconomic position

## Footnotes

1 One-step analysis was performed as a robustness check. It consists of estimating the measurement model using the disability items at baseline and jointly performing a discrete time survival analysis for the 10-year period, without storing factor scores (first step) and then introducing them in the survival model (second step). Both analyses returned very similar results, therefore, for practical reasons only the results from the two-step analysis are reported here (results from the one step analysis available from corresponding author).

2 Second-order model had a good fit (CFI = 0.945, TLI = 0.942, RMSEA = 0.042), but presented some problems: activity measured disability very poorly and its factor loading had an extreme value and was not significant (28.2 and 95 % CI [−120.3, 176.8]; *p* value = 0.71). At wave 2, the value was even more extreme and the model did not converge.

3 Maximum likelihood estimator would have been too cumbersome given the large number of dimensions to be integrated.

4 *Hazard probability* is the term used in Muthen's and Masyn’s paper. The authors defined *the sample-estimated hazard probability for time period j as the number of events that are observed to occur in time period j divided by the total number of subjects at risk in time period j* (p. 33). In the context of our analysis, we will also be using the term *mortality risk* instead of hazard probability.

5 In a general latent variable framework, the likelihood for a latent class model with binary indicators gives the probability of the event indicator being equal to one; in Mplus it is a (negative) “threshold” which defines the cut-point in the latent variable distribution for the switch from ‘category’ 0–1, and it is estimated for each time interval (i.e. her we estimate ten thresholds).

6 The notation is the one used in Muthen and Masyn [17].
