# Supplementary material for: Disability and all-cause mortality in the older population: evidence from the English Longitudinal Study of Ageing
Source: Eur J Epidemiol. 2016 May 13;31(8):735–46. doi: 10.1007/s10654-016-0160-8 (PMC5005412; doi:10.1007/s10654-016-0160-8)
Supplement: Supplementary file 1 — Supplementary material 1 (DOCX 39 kb) [file 10654_2016_160_MOESM1_ESM.docx]

**Supplementary Material A: Tables**

**Supplementary Table 1.** **Disability Items agreed by three authors**

| (1) Impairment | | |
| --- | --- | --- |
| Has a doctor ever told you that you have (or have had) any of the conditions on this card? | High blood pressure or hypertension |  |
|  | Arthritis (including osteoarthritis , or rheumatism) |  |
|  | Parkinson's disease |  |
|  | Any emotional, nervous or psychiatric problems |  |
|  | Dementia, organic brain syndrome, senility or any other serious memory impairment |  |
| Is your eyesight (using glasses or corrective lens as usual) [excellent, very good, good, fair, poor, blind] | | |
| How good is your eyesight for seeing things at a distance, like recognising a friend across the street (using glasses or corrective lens as usual)? [excellent, very good, good, fair, poor, blind] | | |
| How good is your eyesight for seeing things up close, like reading ordinary newspaper print (using glasses or corrective lens as usual)? [excellent, very good, good, fair, poor, blind] | | |
| Is your hearing (using a hearing aid as usual) [excellent, very good, good, fair, poor] | | |
| Are you often troubled with pain? | | |
| How often do you have problems with dizziness when you are walking on a level surface? [never, sometimes, often, very often, always, no walk] | | |
| Have you ever had a severe pain across the front of your chest lasting for half an hour or more? | | |
| Have you ever had any pain or discomfort in your chest? | | |
| Are you troubled by shortness of breath when hurrying on level ground or walking up a slight hill? | | |
| Have you ever had attacks of shortness of breath with wheezing? | | |
| This might not be easy to talk about, but we would like to ask you about incontinence. During the last 12 months, have you lost any amount of urine beyond your control? | | |
| How would you rate your memory at the present time? [excellent, very good, good, fair, poor] | | |
| Do you get pain or discomfort in either of your legs which comes on when you walk? [no, yes, can't walk] | | |
| Much of the time during the past week, you felt depressed? | | |

(2) Activity

| *Please tell me whether you have any difficulty doing each of the everyday activities on this card. Exclude any difficulties that you expect to last less than three months. Because of a health problem, do you have difficulty doing any of the activities on this card?* | Walking 100 yards |
| --- | --- |
|  | Sitting for about two hours |
|  | Getting up from a chair after sitting for long periods |
|  | Climbing several flights of stairs without resting |
|  | Climbing one flight of stairs without resting |
|  | Stooping, kneeling, or crouching |
|  | Reaching or extending your arms above shoulder level |
|  | Pulling or pushing large objects like a living room chair |
|  | Lifting or carrying weights over 10 pounds |
|  | Picking up a 5p coin from a table |
| *Please tell me if you have any difficulty with these because of a physical, mental, emotional or memory problem. Again exclude any difficulties you expect to last less than three months. Because of a health or memory problem, do you have difficulty doing any of the activities on this card?* | Dressing, including putting on shoes and socks |
|  | Walking across a room |
|  | Bathing or showering |
|  | Eating, such as cutting up your food |
|  | Getting in or out of bed |
|  | Using the toilet, including getting up or down |
| Do you find it difficult to follow a conversation if there is background noise | |
| How often do you have problems with keeping your balance when you are walking on a level surface? [never, sometimes, often, very often, always, no walk] | |
| By yourself and without using any special equipment, how much difficulty do you have walking for a quarter of a mile? [no difficulty, some difficulty, much difficulty, unable] | |
| (Much of the time during the past week), your sleep was restless? | |

(3) Participation

| *Please tell me if you have any difficulty with these because of a physical, mental, emotional or memory problem. Again exclude any difficulties you expect to last less than three months. Because of a health or memory problem, do you have difficulty doing any of the activities on this card?* | Preparing a hot meal | |
| --- | --- | --- |
|  | Using a map to figure out how to get around in a strange place | |
|  | Shopping for groceries | |
|  | Making telephone calls | |
|  | Doing work around the house or garden | |
|  | Managing money, such as paying bills and keeping track of expenses | |
| Do you use public transport? If no Why don't you use public transport more often?[My health prevents me] | | |
| What were your reasons for retiring? [Own ill health] | | |
| What were your reasons for taking early retirement? [Own ill health] | | |

**Supplementary Table** **2.** **Missing Data Pattern**

1. Patterns

| N | Marital status | Alcohol | Chronic illness | Physical activity | Education | Ethnicity | Father's job | Income | Wealth | Occupation |
| --- | --- | --- | --- | --- | --- | --- | --- | --- | --- | --- |
| 9295 | 1 | 1 | 1 | 1 | 1 | 1 | 1 | 1 | 1 | 1 |
| 155 | 1 | 1 | 1 | 1 | 1 | 1 | 1 | 1 | 1 | 0 |
| 140 | 1 | 1 | 1 | 1 | 1 | 1 | 1 | 0 | 0 | 1 |
| 106 | 1 | 1 | 1 | 1 | 1 | 1 | 0 | 1 | 1 | 1 |
| 6 | 1 | 1 | 1 | 1 | 1 | 0 | 1 | 1 | 1 | 1 |
| 2 | 1 | 0 | 1 | 1 | 1 | 1 | 1 | 1 | 1 | 1 |
| 2 | 1 | 1 | 0 | 1 | 1 | 1 | 1 | 1 | 1 | 1 |
| 2 | 1 | 1 | 1 | 1 | 0 | 1 | 1 | 1 | 1 | 1 |
| 2 | 1 | 1 | 1 | 1 | 1 | 1 | 0 | 0 | 0 | 1 |
| 1 | 0 | 1 | 1 | 1 | 1 | 1 | 1 | 1 | 1 | 1 |
| 1 | 1 | 1 | 1 | 0 | 1 | 1 | 0 | 1 | 1 | 1 |
| 1 | 1 | 1 | 1 | 0 | 1 | 1 | 1 | 1 | 1 | 1 |
| 1 | 1 | 1 | 1 | 1 | 0 | 1 | 0 | 1 | 1 | 1 |
| 1 | 1 | 1 | 1 | 1 | 1 | 1 | 1 | 0 | 0 | 0 |

**Supplementary Table 3.** **DTSA using FIML**

|  | Time since disability measurement | OR | 95% CI |
| --- | --- | --- | --- |
| MALES | 1 | 2.271*** | (1.15,3.39) |
|  | 2 | 1.679*** | (1.09,2.27) |
|  | 3 | 1.85*** | (1.27,2.43) |
|  | 4 | 1.389** | (1.02,1.76) |
|  | 5 | 1.485*** | (1.08,1.89) |
|  | 6 | 1.01 | (0.73,1.29) |
|  | 7 | 1.344* | (0.95,1.74) |
|  | 8 | 1.315* | (0.95,1.68) |
|  | 9 | 1.412*** | (1.07,1.76) |
|  | 10 | 1.916*** | (1.34,2.49) |
| FEMALES | time-invariant effect | 1.365*** | (1.2,1.53) |

**Supplementary Table 4.** **Sample desc****riptive statistics**

|  | Males (N=4,455) | | Females (N=5,260) | |
| --- | --- | --- | --- | --- |
| *Variables* | N | Prevalence | N | Prevalence |
| Age |  |  |  |  |
| 50-64 | 2,402 | 53.9 | 2,791 | 53.1 |
| 65-74 | 1,278 | 28.7 | 1,446 | 27.5 |
| 75+ | 775 | 17.4 | 1,023 | 19.5 |
| *Number of deaths ^a^* | 940 | 21.9 *^a^* | 835 | 16.5 *^a^* |
| *Marital status* | |  |  |  |
| single | 262 | 5.9 | 253 | 4.8 |
| married | 3,434 | 77.1 | 3,217 | 61.2 |
| divorced or separated | 378 | 8.5 | 614 | 11.7 |
| widowed | 380 | 8.5 | 1,176 | 22.4 |
| Total | 4,454 | 100 | 5,260 | 100 |
| *Parental status* | |  |  |  |
| Yes | 3,460 | 77.7 | 4,267 | 81.1 |
| No | 995 | 22.3 | 993 | 18.9 |
| Total | 4,455 | 100 | 5,260 | 100 |
| *Household size* | 4,455 | 2.17 | 5,260 | 1.93 |
| *Ethnicity* |  |  |  |  |
| White | 4,338 | 97.4 | 5,166 | 98.3 |
| non-White | 114 | 2.6 | 91 | 1.7 |
| Total | 4,452 | 100 | 5,257 | 100 |
| *Education* |  |  |  |  |
| high educate | 1,303 | 29.3 | 976 | 18.6 |
| middle educated | 1,632 | 36.7 | 1,952 | 37.1 |
| low educated | 1,518 | 34.1 | 2,331 | 44.3 |
| Total | 4,453 | 100 | 5,259 | 100 |
| *Income* |  |  |  |  |
| 1st quintile | 683 | 15.5 | 1,232 | 23.8 |
| 2nd quintile | 845 | 19.2 | 1,069 | 20.7 |
| 3rd quintile | 911 | 20.7 | 1,004 | 19.4 |
| 4th quintile | 976 | 22.2 | 938 | 18.2 |
| 5th quintile | 988 | 22.4 | 926 | 17.9 |
| Total | 4,403 | 100 | 5,169 | 100 |
| *Wealth* |  |  |  |  |
| 1st quintile | 818 | 18.6 | 1,097 | 21.2 |
| 2nd quintile | 860 | 19.5 | 1,055 | 20.4 |
| 3rd quintile | 882 | 20 | 1,032 | 20 |
| 4th quintile | 914 | 20.8 | 1,001 | 19.4 |
| 5th quintile | 929 | 21.1 | 984 | 19 |
| Total | 4,403 | 100 | 5,169 | 100 |
| *Occupation* |  |  |  |  |
| Managerial or professional | 1,529 | 34.4 | 1,076 | 21 |
| intermediate | 1,441 | 32.5 | 2,004 | 39.2 |
| Routine or technical | 1,470 | 33.1 | 2,039 | 39.8 |
| Total | 4,440 | 100 | 5,119 | 100 |
| *Father's job when respondent was 14* |  |  |  |  |
| professional or managerial | 1,240 | 28.1 | 1,594 | 30.7 |
| skilled | 1,284 | 29.1 | 1,427 | 27.5 |
| unskilled | 598 | 13.6 | 698 | 13.4 |
| other | 1,286 | 29.2 | 1,478 | 28.4 |
| Total | 4,408 | 100 | 5,197 | 100 |
| Smoking |  |  |  |  |
| never | 1,166 | 26.2 | 2,315 | 44 |
| ex-smoker | 2,530 | 56.8 | 2,038 | 38.8 |
| current | 759 | 17 | 907 | 17.2 |
| Total | 4,455 | 100 | 5,260 | 100 |
| *Drinking* |  |  |  |  |
| heavy drinker | 260 | 5.8 | 167 | 3.2 |
| normal drinker | 3,330 | 74.8 | 3,102 | 59 |
| non-drinker | 863 | 19.4 | 1,991 | 37.9 |
| Total | 4,453 | 100 | 5,260 | 100 |
| *Physical activity* | |  |  |  |
| very active | 2,987 | 67.1 | 3,289 | 62.5 |
| normally active | 1,161 | 26.1 | 1,602 | 30.5 |
| inactive | 306 | 6.9 | 368 | 7 |
| Total | 4,454 | 100 | 5,259 | 100 |
| *Long-lasting illness* | |  |  |  |
| Yes | 2,480 | 55.7 | 2,917 | 55.5 |
| No | 1,975 | 44.3 | 2,341 | 44.5 |
| Total | 4,455 | 100 | 5,258 | 100 |
| *Inflammation* | 2,494 | 4.04 | 2,919 | 3.97 |
| *Coagulation* | 2,482 | 3.16 | 2,900 | 3.26 |
| *Lung function* | 2,951 | 3.8 | 3,494 | 2.65 |
| *Cholesterol* | 2,497 | 5.62 | 2,921 | 6.18 |

^a^ Percentages estimated on the total number of individual whose mortality status was available

**Supplementary Table 5. First or****der measurement model’s factor loadings^1^**

(1) Impairment

| Impairment | Estimate (S.E.) |
| --- | --- |
| Hypertension | 0.24 (0.015) |
| **Arthritis** | **0.615 (0.011)** |
| Parkinson | 0.375 (0.048) |
| Emotional or psychiatric problems | 0.219 (0.021) |
| Dementia | 0.377 (0.058) |
| Hearing | 0.315 (0.011) |
| Sight | 0.366 (0.011) |
| Sight at distance | 0.357 (0.011) |
| Sight close | 0.356 (0.011) |
| **Pain** | **0.727 (0.009)** |
| **Chest-pain** | **0.454 (0.013)** |
| **Pain across the front of chest** | **0.467 (0.017)** |
| **Pain in legs** | **0.752 (0.008)** |
| **Dizziness** | **0.72 (0.011)** |
| **Shortness of breath** | **0.781 (0.007)** |
| **Breath with wheezing** | **0.436 (0.016)** |
| **Incontinence** | **0.439 (0.016)** |
| Memory problems | 0.251 (0.011) |
| **Depression** | **0.439 (0.015)** |

(2) Eyesight

| Eyesight | Estimate (S.E.) |
| --- | --- |
| **Sight** | **0.808 (0.005)** |
| **Sight at distance** | **0.774 (0.006)** |
| **Sight close** | **0.793 (0.005)** |

(3) Activity

| Activity | Estimate (S.E.) |
| --- | --- |
| **Walking 100 yards** | **0.937 (0.005)** |
| **Sitting for about 2 hours** | **0.686 (0.011)** |
| **Getting up from a chair** | **0.774 (0.008)** |
| **Climbing several flights of stairs** | **0.837 (0.006)** |
| **Climbing one flight of stairs** | **0.889 (0.006)** |
| **Stooping- kneeling or crouching** | **0.796 (0.007)** |
| **Reaching or extending arms above shoulder** | **0.693 (0.012)** |
| **Pulling or pushing large objects** | **0.877 (0.006)** |
| **Lifting or carrying weights over 10 pounds** | **0.862 (0.006)** |
| **Picking up a 5p coin from a table** | **0.644 (0.017)** |
| **Getting dressed** | **0.801 (0.009)** |
| **Walking across room** | **0.875 (0.011)** |
| **Bathing** | **0.851 (0.008)** |
| **Eating** | **0.697 (0.021)** |
| **Getting in and out of bed** | **0.826 (0.01)** |
| **Toileting** | **0.766 (0.015)** |
| Following conversation | 0.31 (0.014) |
| **Balance** | **0.79 (0.007)** |
| **Walking quarter of mile** | **0.922 (0.004)** |
| **Restless sleep** | **0.416 (0.013)** |

(4) Participation

| Participation | Estimate (S.E.) |
| --- | --- |
| **Preparing a hot meal** | **0.874 (0.011)** |
| **Using a map to get oriented** | **0.538 (0.021)** |
| **Shopping for groceries** | **0.926 (0.007)** |
| **Making telephone calls** | **0.527 (0.029)** |
| **Doing work around house or garden** | **0.935 (0.006)** |
| **Managing money** | **0.615 (0.025)** |
| **Using public transport** | **0.885 (0.009)** |
| Being member of any organization | 0.262 (0.015) |
| **Engaging in any activities** | **0.474 (0.014)** |
| Early retirement due to health problems | 0.324 (0.022) |
| Retirement due to health problems | 0.385 (0.023) |

^1^Factor loadings larger than 0.4 are reported in bold

**Supplementary Table 6. Likelihood Ratio Test**

| Variable | Males | | | Females | | |
| --- | --- | --- | --- | --- | --- | --- |
|  | LR Chi square | df | p-value | LR Chi square | df | p-value |
| Disability | 24.46 | 9 | 0.0036 | 11.21 | 9 | 0.2619 |
|  | 17.02^a^ | 9 | 0.0484 | 7.84 ^a^ | 9 | 0.5507 |
| Age | 23.86 | 9 | 0.0045 | 26.58 | 9 | 0.0016 |
| Marital status | 33.52 | 27 | 0.1804 | 19.85 | 27 | 0.837 |
| Household size | 11.26 | 9 | 0.2584 | 9 | 9 | 0.4372 |
| Parental status | 9.55 | 9 | 0.388 | 9.17 | 9 | 0.4217 |
| Ethnicity | 3.63 | 6 | 0.7267 | 0.78 | 3 | 0.8536 |
| Education | 9.27 | 18 | 0.9531 | 18.97 | 18 | 0.3936 |
| Occupation | 7.96 | 18 | 0.9792 | 22.18 | 18 | 0.224 |
| Income | 36.52 | 36 | 0.4445 | 26.57 | 35 | 0.8464 |
| Wealth | 19.52 | 36 | 0.9885 | 25.8 | 36 | 0.896 |
| Smoking | 19.28 | 18 | 0.3749 | 12.76 | 18 | 0.8057 |
| Drinking | 14.3 | 18 | 0.7097 | 16.1 | 17 | 0.5169 |
| Physical activity | 47.65 | 18 | 0.0002 | 24.19 | 18 | 0.149 |
| Father's job | 20.52 | 27 | 0.8081 | 25.02 | 27 | 0.5733 |
| Chronic illness | 12.38 | 9 | 0.1928 | 6.74 | 9 | 0.664 |
| ^a^ from full model | | | | | | |

**Supplementary Table 7. DTSA by sex and age group using disability estimated via multiple group analysis by age group**

|  |  | 50-64 | | 65-74 | | 75+ | |
| --- | --- | --- | --- | --- | --- | --- | --- |
|  | Time since disability measurement | OR | 95% CI† | OR | 95% CI† | OR | 95% CI† |
| MALES | 1 | 3.622** | (1.11;11.83) | 4.076** | (1.1;15.1) | 1.283 | (0.63;2.61) |
|  | 2 | 1.714 | (0.71;4.16) | 1.696* | (0.91;3.14) | 1.741*** | (1.15;2.65) |
|  | 3 | 2.232** | (1.2;4.16) | 1.928*** | (1.2;3.1) | 1.616** | (1.02;2.57) |
|  | 4 | 1.604** | (1;2.57) | 1.413 | (0.87;2.28) | 1.132 | (0.75;1.71) |
|  | 5 | 1.405 | (0.8;2.47) | 1.507* | (0.95;2.4) | 1.34 | (0.89;2.01) |
|  | 6 | 0.971 | (0.48;1.95) | 0.866 | (0.55;1.36) | 1.252 | (0.8;1.97) |
|  | 7 | 1.894** | (1.04;3.43) | 1.19 | (0.73;1.94) | 1.129 | (0.75;1.7) |
|  | 8 | 2.156*** | (1.23;3.77) | 1.147 | (0.74;1.78) | 1.058 | (0.69;1.62) |
|  | 9 | 1.267 | (0.73;2.21) | 1.488* | (0.95;2.33) | 1.354 | (0.87;2.1) |
|  | 10 | 2.081*** | (1.26;3.44) | 1.632* | (1;2.67) | 2.07*** | (1.24;3.45) |
| FEMALES | time-invariant effect | 1.580*** | (1.2;2.08) | 1.286** | (1.04;1.59) | 1.267*** | (1.08;1.48) |

† SE estimated from pooled logistic regression

**Supplementary Table 8. DTSA using disability measurement by sex and via multiple group analysis by sex**

|  |  | (1) | | (2) | |
| --- | --- | --- | --- | --- | --- |
|  | Time since disability measurement | OR | 95% CI† | OR | 95% CI† |
| MALES | 1 | 2.351*** | (1.29; 4.28) | 2.341*** | (1.3; 4.22) |
|  | 2 | 1.824*** | (1.28; 2.61) | 1.836*** | (1.29; 2.62) |
|  | 3 | 1.966*** | (1.43; 2.7) | 1.945*** | (1.42; 2.66) |
|  | 4 | 1.472*** | (1.11; 1.94) | 1.457*** | (1.11; 1.92) |
|  | 5 | 1.479*** | (1.11; 1.97) | 1.477*** | (1.11; 1.97) |
|  | 6 | 1.04 | (0.76; 1.42) | 1.039 | (0.76; 1.41) |
|  | 7 | 1.325* | (0.99; 1.78) | 1.319* | (0.98; 1.77) |
|  | 8 | 1.317* | (0.99; 1.75) | 1.302* | (0.98; 1.73) |
|  | 9 | 1.406** | (1.06; 1.87) | 1.395** | (1.05; 1.86) |
|  | 10 | 2.013*** | (1.48; 2.73) | 1.989*** | (1.47; 2.69) |
| FEMALES | time-invariant effect | 1.357*** | (1.21 ; 1.53) | 1.364*** | (1.21; 1.54) |
| (1) fully adjusted model with factor scores estimated separately by gender | | | | | |
| (2) fully adjusted model with factor scores estimated using multi-group analysis  † SE estimated from pooled logistic regression | | | | | |

**Supplementary Table 9. DTSA using disability measurement not including chronic conditions**

|  |  | (1) | | (2) | |
| --- | --- | --- | --- | --- | --- |
|  | Time since disability measurement | OR | 95% CI† | OR | 95% CI† |
| MALES | 1 | 2.19*** | (1.24,3.85) | 2.14*** | (1.21,3.78) |
|  | 2 | 1.8*** | (1.28,2.54) | 1.76*** | (1.24,2.49) |
|  | 3 | 1.87*** | (1.38,2.53) | 1.82*** | (1.34,2.47) |
|  | 4 | 1.44*** | (1.1,1.88) | 1.4** | (1.07,1.84) |
|  | 5 | 1.46*** | (1.1,1.93) | 1.42** | (1.07,1.88) |
|  | 6 | 1.04 | (0.77,1.41) | 1.01 | (0.74,1.37) |
|  | 7 | 1.33* | (0.99,1.77) | 1.28* | (0.96,1.72) |
|  | 8 | 1.32** | (1,1.75) | 1.28* | (0.97,1.69) |
|  | 9 | 1.38** | (1.04,1.82) | 1.33** | (1,1.77) |
|  | 10 | 1.96*** | (1.45,2.64) | 1.9*** | (1.41,2.56) |
| FEMALES | time-invariant effect | 1.39*** | (1.23,1.56) | 1.38*** | (1.23,1.56) |
| (1) fully adjusted model | |  |  |  |  |
| (2) fully adjusted model controlling also for chronic conditions | | | |  |  |
| † SE estimated from pooled logistic regression | | |  |  |  |

**Supplementary Material B**

**Rationale for choosing general-specific model**

Disability was conceived as a general independent factor, and impairment, activity and participation as separate specific factors. This means that disability was assumed to explain the part of variation of the observable items that was not captured by specific factors, and it was assumed not to be correlated with them. The general-specific model differs from higher-order models in that first-order factors are not subsumed by the higher order factor but are, instead, uncorrelated and distinct. A second-order model would have appeared to be more coherent with the WHO’s conceptualization of disability, but in fact the choice of a general-specific model had both theoretical and empirical advantages. General-specific models are potentially applicable when (a) there is a general factor that is hypothesized to account for the commonality of the items; (b) there are multiple domain specific factors, each of which is hypothesized to account for the unique influence of the specific domain over and above the general factor; (c) there may be an interest in the domain specific factors as well as the common factor that is of focal interest (Cheng et al. [45], p.190). The last two points in particular, we believe, justify our choice of applying a general-specific model. As a matter of fact, the innovative and fundamental feature of the ICF is that it allows us to disentangle the various disability components in order to estimate separately their impact on different spheres. The empirical foundation for preferring a general-specific model was that in the multidimensional first order model that we firstly estimated, we observed that impairment (I), activity (A) and participation (P) were very highly correlated (A with I=0.989; A with P=0.988; I with P=0.963), whilst the eyesight factor was very specific (correlation of eyesight factor with I=0.075; with A=0.051; with P=0.049). This means that the eyesight factor captured some variance which was unique to its specific items, i.e. factor loadings of eye items to eyesight factor remained high, implying that there was a very strong specific variance that was not related to disability. For all these reasons a general-specific model was preferred to a second-order model.
